# Prophylactic endoscopic pylorus dilatation prior to esophagectomy for esophageal cancer to prevent delayed gastric emptying, study protocol for a placebo-controlled randomized trial (PROPPER trial)

**DOI:** 10.1186/s13063-025-08912-9

**Published:** 2025-06-23

**Authors:** C. Mann, F. Berlth, V. J. Lozanovski, M. Passalacqua, E. Hadzijusufovic, E. Uzun, G. Capovilla, M. Valmasoni, H. Lang, P. P. Grimminger

**Affiliations:** 1 Department of General, Visceral, and Transplantation Surgery, University Medical Center Mainz, Mainz, Germany; 2 Department of General, Visceral, and Transplant Surgery, University Hospital Tübingen, Tübingen, Germany; 3 Center for Esophageal Diseases - Department of Surgical, Oncological and Gastroenterological Sciences, University of Padova, Padua, Italy

**Keywords:** Ivor–Lewis esophagectomy, Gastric emptying, Gastric pull-up, Minimally invasive esophagectomy, Pneumatic dilatation

## Abstract

**Introduction:**

Delayed gastric emptying (DGE) due to pyloric dysfunction remains a common postoperative complication after esophagectomy for cancer and can lead to severe secondary complications. As shown in a retrospective study, prophylactic EPBD performed 1 day before surgery can reduce the rate of postoperative DGE by reducing pyloric resistance.

The objective of this study is to analyze the effect of prophylactic EPBD on postoperative DGE rates in patients receiving minimally invasive esophagectomy for cancer by gastric pull-up.

**Methods:**

This study is designed as a multicenter randomized controlled trial (RCT) including patients with esophageal cancer or cancer of the gastroesophageal junction (adenocarcinoma and squamous cell carcinoma, with or without neoadjuvant treatment) scheduled for minimally invasive esophagectomy with gastric pull-up. After randomization, patients will either receive preoperative EPBD or a sham intervention in the routine preoperative endoscopy performed 1 day before surgery. The primary endpoint of this study will be rates of DGE, particularly those resulting from pyloric dysfunction, requiring intervention. Secondary outcomes will be major and minor postoperative complication rates, in-hospital mortality, adverse events during gastroscopy, length of ICU and hospital stay as well as postoperative pain and quality of life. In order to detect a difference between both groups at a two-sided 5% significance level, to achieve a power of 0.8 with a calculated dropout rate of approximately 20%, a sample size of 118 patients with 59 patients in every study arm will be needed.

**Discussion:**

The presented PROPPER trial is the first multicenter RCT that will provide evidence regarding the efficacy of preoperative EPBD in reducing DGE after minimally invasive esophagectomy for cancer.

**Trial registration:**

This trial was registered in the German Clinical Trials Register (DRKS), under the identifier DRKS00034360. Registered on May 29, 2024.

The WHO trial registration data set can be found here: http://drks.de/search/en/trial/DRKS00034360.

**Supplementary Information:**

The online version contains supplementary material available at 10.1186/s13063-025-08912-9.

## Introduction

Esophageal cancer (EC) with worldwide estimated 604,000 new cases alone in 2020 is the sixth leading cause of death from cancer [1]. Radical transthoracic esophagectomy with 2-field lymphadenectomy remains a crucial part of the multimodal approach to treat esophageal cancer or cancer of the gastroesophageal junction [2].

Due to the improvement of surgical strategies and perioperative management, as well as better patient selection, postoperative morbidity after esophagectomy could be reduced in recent years [3]. However, postoperative delayed gastric emptying (DGE) caused by pyloric dysfunction still affects approximately 10–50% of patients [4]. In further course, DGE can lead to additional complications, such as aspiration, pneumonia and prolonged hospital stay. Causes for postoperative DGE are multifactorial and include truncal vagotomy performed during the resection, the new position of the stomach in a negative pressure environment in the thorax, as well as possible relative hypoxia of the conduit leading to decelerated peristalsis [5, 6]. Although postoperative DGE due to pyloric dysfunction can be safely and effectively treated by endoscopic pyloric balloon dilatation (EPBD) [7], secondary complications are present in many patients at the time of treatment. Thus, a prophylactic approach to avoid DGE in the first place is of utmost interest.

Potential alternatives to preoperative EPBD are maneuvers such as endoscopic botulinum toxin injection or surgical pyloromyotomy [8–10]. The latest meta-analysis could not affirm the efficacy of botulinum toxin injection on postoperative DGE rates [11, 12]. Surgical pyloric drainage, such as pyloroplasty or pyloromyotomy, might be accompanied by procedure-specific complications, including bile reflux, gastric dumping, and leakage. Furthermore, the systematic review and meta-analysis by Nevins et al. showed no significant effect of intra-operative gastric drainage procedures on postoperative DGE rates [13]. Most surgeons consequently omitted these procedures over time. Nienhüser et al. presented a meta-analysis in 2022, including four retrospective studies that analyzed the efficacy of mechanical stretching with a significant reduction in the postoperative DGE rate [9]. Three of those studies performed mechanical stretching intraoperatively, either manually or using a forceps. However, these maneuvers are not practicable in times of minimally invasive surgery. As the fourth study included in the meta-analysis, Hadzijusufovic et al. proposed preoperative EPBD to prevent postoperative DGE in 2019 [14]. In this retrospective study, 91 patients were treated with preoperative EPBD 1 day before scheduled esophagectomy with Ivor-Lewis reconstruction (pyloric dilatation group, PDG) and the postoperative complication rates were compared to 24 patients not receiving preoperative EPBD (non pyloric dilatation group, NPDG). Significantly fewer patients in the PDG suffered from postoperative DGE (13.2%) compared to the NPDG (37.5%, *p* = 0.014), measured by required postoperative EPBD. These retrospective results imply that preoperative EPBD can prevent the occurrence of postoperative DGE in patients treated with Ivor-Lewis esophagectomy. However, the mentioned study has several limitations, including the small sample size of 115 patients, as well as a possible selection bias since the intervention group was not allocated by randomization but by external circumstances such as stenotic tumor or logistic reasons. Therefore, prospective evidence regarding the efficacy of preoperative endoscopic pyloric dilatation is lacking, and a randomized controlled trial is needed. This RCT is the first to evaluate whether prophylactic preoperative EPBD reduces postoperative DGE. This could change standard preoperative treatment of patients receiving minimally invasive esophagectomy for cancer and significantly reduce postoperative complication rates.

## Methods/design

### Study objectives

The aim of the PROPPER trial is to compare, in a prospective randomized setting, the impact of preoperatively performed EPBD in patients with resectable intrathoracic esophageal cancer or cancer of the gastroesophageal junction versus a control group receiving a placebo intervention. The primary objective is to determine whether preoperative EPBD reduces postoperative pyloric dysfunction after minimally invasive thoracoabdominal esophagectomy.

### Study design

The PROPPER trial is a multicenter, two parallel-group randomized controlled clinical superiority trial. The “Standard Protocol Items: Recommendations for Interventional Trials” (SPIRIT) 2013 statement was used to structure the design of this study [15] (Appendix 1). The study will be conducted in three tertiary referral clinics and university hospitals carrying out esophageal cancer resections:

1. Department of General, Visceral, and Transplantation Surgery, University Medical Center Mainz
2. Department of General, Visceral, and Transplant Surgery, University Hospital Tübingen, Center for Esophageal Diseases
3. Department of Surgical, Oncological, and Gastroenterological Sciences—University of Padova

Patients presenting with resectable esophageal cancer or cancer of the gastroesophageal junction are assessed for meeting the inclusion or exclusion criteria (Table 1). After counseling by a treating surgeon, written consent of all participating patients will be obtained.

**Table 1:** Inclusion and exclusion criteria

**Inclusion criteria**
Histological proven squamous cell carcinoma or adenocarcinoma of the intrathoracic esophagus or gastroesophageal junction, planned as minimally invasive esophagectomy (RAMIE/MIE) with Ivor-Lewis reconstruction
Surgical resectability, cT1-4a, cNx, cM0
Age $$\ge$$ 18 and $$\le$$ 80
ECOG performance status 0–2
Written consent
**Exclusion criteria**
Carcinoma of the cervical esophagus
Functional inoperability
Previous surgery of the stomach or esophagus
Esophageal stenosis at time of potential study inclusion

### Primary and secondary endpoints

The primary endpoint of the PROPPER trial is the occurrence rate of pyloric dysfunction. Pyloric dysfunction is diagnosed if one of the following symptoms appears during the first 14 days after surgery:

1. One-time vomiting of either liquid or sieved food during gradual return to oral food intake
2. Recurrent vomiting of solid food during gradual return to oral food intake
3. Performed EPBD independent of vomiting or oral food intake for other reasons

(Oral food intake has to be initiated latest 10 days after surgery to evaluate postoperative pyloric dysfunction.)

This definition was elected since vomiting during the process of returning to oral food intake after esophagectomy is the one clinical parameter that indicates pyloric dysfunction. However, clinical experience shows that one-time vomiting after solid food intake happens occasionally after overeating and dietary errors. Therefore, vomiting after solid food intake must happen repeatedly to count as pyloric dysfunction. In all participating centers, postoperative vomiting, as defined above, triggers performing endoscopy and EPBD.

The timing of 14 days after surgery was chosen in accordance with the international expert consensus based on a modified Delphi process published in 2020 [16]. DGE that occurs within 14 days after the surgery was defined as early DGE. We suspect early DGE to be affected most by preoperative EPBD since the time between intervention and outcome is short, and after weeks or months, additional disturbance variables such as other complications might add bias to our results.

The primary outcome is assessed by study nurses responsible for data acquisition. They will interview the patients about any vomiting after the surgery. Thereby, we ensure to receive honest answers and capture patient experience.

### Secondary endpoints

Secondary outcome parameters include major and minor postoperative complication rates (Clavien-Dindo ≥ 3b and ≤ 3a), in hospital mortality, adverse events during gastroscopy, length of ICU and hospital stay as well as postoperative pain, measured by visual analog scale (VAS), quality of life, as measured by SF-36, EORTC QLQ-C30, EORTC OES18, and EQ-5D questionnaires and overall survival. Pain and QoL will be assessed at four time points: preoperatively, within 5 days post-op, at 4 weeks, and at 3 months.

### Study population

All adult patients (age 18–80 years) with histologically proven squamous cell carcinoma or adenocarcinoma of the esophagus or the gastroesophageal junction and surgical resectability (T1-4a, N0–3, M0) will be assessed for participation in the study. Only patients with European Clinical Oncology Group (ECOG) performance status 0–2 and planned intrathoracic anastomosis are eligible for the study. Inclusion and exclusion criteria are listed in Table 1. Patients with known esophageal stenosis cannot receive the planned intervention since the endoscope needs to reach the pylorus for EPBD and are thus excluded from the study. The patient will be blinded throughout the whole study.

### Ethics

This study is conducted in accordance with the principles of the Declaration of Helsinki. The independent ethics committee of Rhine-Land-Palatinate, Germany has approved the study protocol (IRB number: 2023–16937, Appendix 2). The decision of the ethics committee in Italy is still pending. Written consent is obtained by all patients prior to randomization (Appendix 3).

### Study protocol

#### Recruitment of research population

Patients are recruited for the study in the outpatients department during their consultation with their treating surgeons, who will obtain informed consent. After giving their consent, all patients have at least 1 week to reconsider their participation.

The recruitment should take approximately 2 years. Total duration of the study will be approximately 7 years. In case of any changes in the protocol, the investigators, clinical research assistants, the ethics committee, and the sponsor are immediately informed by email.

#### Randomization and blinding methods

Patients will be randomized at the outpatient department to either PDG or NPDG. Randomization is executed centrally by an online randomization program for each participating center by the responsible study nurse. The online randomization program can be accessed by all centers via a personal log in on a website (propper-trial.goip.de). It was set up by independent statisticians who did not participate in the trial. The program is designed to allow only a certain number of randomization processes. The study nurse executing the randomization will inform the executing endoscopist about the randomization status on the day of the intervention.

While patients are blinded to the intervention, medical personnel (surgeons, endoscopists) are not, due to safety reasons. If patients show any symptoms or change in their clinical course after endoscopy, the treating doctors and nurses should be aware if the pneumatic dilatation was performed. On the one hand, because the intervention itself could cause a complication such as bleeding or perforation; on the other hand, other symptoms could falsely be explained by a dilatation. Therefore, we decided to unblind all treating personnel. However, the study nurses that will collect the outcomes from the patients and are responsible for data acquisition are unaware of the randomization status.

If any unintentional unblinding occurs, the study coordinator will be contacted.

#### Pylorus dilatation/EPBD

All patients will routinely receive a gastroscopy the day before surgery to confirm intraluminal tumor extent and exact location after possible neoadjuvant treatment. After neoadjuvant treatment tumor size and consequently surgical strategy could change. Therefore, it is essential that a patient consultation after endoscopy and before surgery is possible. The gastroscopy is performed under analgosedation with propofol. In patients randomized to the PDG, a 2-min balloon dilatation of the pylorus with a 20-mm balloon (Boston Scientific, Ireland) will be performed. This dilatation is performed through the scope by water insufflation with a pressure of 608 kPa. Patients randomized to the NPDG receive staging endoscopy without pylorus dilatation. If—due to any reason (e.g., stenotic tumor)—balloon dilatation is not possible, it will not be carried out during the preoperative gastroscopy. The patient can request to terminate the study participation at any time. The intervention is proven to be a feasible and safe and part of the clinical routine in all participating centers. The study will impose no additional risk for the included patients.

#### Placebo intervention

The placebo intervention is the gastroscopy without performing EPBD. The pylorus is passed by the endoscope as it is in the intervention group. Gastroscopy 1 day preoperatively is primarily used for restaging and surgical planning. Therefore, no unnecessary examination is carried out in the placebo group.

During the intervention, the patients are sedated. After dilatation, there are no scars or visible changes, nor can the patient sense the difference between the intervention and an ordinary endoscopy. The only possibility for the patients to discover their group allocation might be the duration of the intervention. However, we do not believe medical laypersons to have those insights.

#### Surgery and perioperative management

All patients undergo a minimally invasive esophagectomy with 2-field lymphadenectomy and Ivor-Lewis reconstruction. This includes formation of a gastric conduit and creation of a transthoracic esophagogastrostomy using a circular stapler, as previously described [17, 18]. In order to avoid bias by inhomogeneous treatment pathways and surgical procedures, we ensure all participating centers perform the minimally invasive esophagectomy according to the published papers. All participating surgeons were trained at University Hospital Mainz and routinely apply the same surgical procedure.

The perioperative management in both groups is equal. Postoperative oral food intake is routinely initiated on the third postoperative day, beginning with water in sips. Thereafter, oral intake is increased with sieved food on the fourth and solid food on the fifth postoperative day.

### Statistical analysis

#### Exclusion to primary analysis

The analysis of the primary endpoint will be performed in accordance with the intention to treat principle. First, an intention-to-treat analysis will be carried out, which involves all patients randomized into the study. Second, a modified per-protocol analysis with following patients excluded will be performed:

1. Endoscopically not passable tumors in the staging endoscopy 1 day before surgery (no intervention possible)
2. Initiation of oral food intake later than 10 days postoperatively due to any reason apart from pyloric dysfunction
3. Anastomotic leakage at a time when oral food intake is not completed or 10 days after initiation of oral food intake has not passed.

Patients fulfilling the above-mentioned criteria cannot be evaluated properly regarding the occurrence of postoperative DGE or pyloric dysfunction. Since DGE does not affect anastomotic leakage rates, a bias towards less DGE in the primary analysis due to the exclusion of patients with anastomotic leakage is not likely [19]. However, anastomotic leakages interrupt the regular course of oral food intake, and its influence on the gastric conduit remains unclear. Therefore, a modified per-protocol analysis will be carried out with the patients not excluded from the primary analysis.

#### Sample size calculation

The hypothesis of this study is a lower rate of pyloric dysfunction in patients receiving preoperative EPBD compared to a control group receiving a sham intervention. In order to assess a sample size, the rates of pyloric dysfunction of the retrospective study of Hadzijusufovic et al. were used, since operative techniques and perioperative treatment pathways do not differ from those used in the PROPPER trial. In this study, the pyloric dysfunction rate in the PDG was 24.3% lower than in the NPDG (13.2% vs. 37.5%). With a two-sided 5% significance level, 49 patients per arm will be needed to achieve a power of 0.8 to detect a difference. Compensating for a dropout rate of approximately 20%, a sample size of 118 patients with 59 patients in every study arm will be needed. After 89 patients, an interim analysis will be carried out. The study will be terminated if the superiority of the EPBD intervention has already been proven or cannot be proven at all. Otherwise, recruitment will be carried on until 118 patients are included. This will be decided by the principal investigator (PI).

#### Missing data

**Fig. 1:**
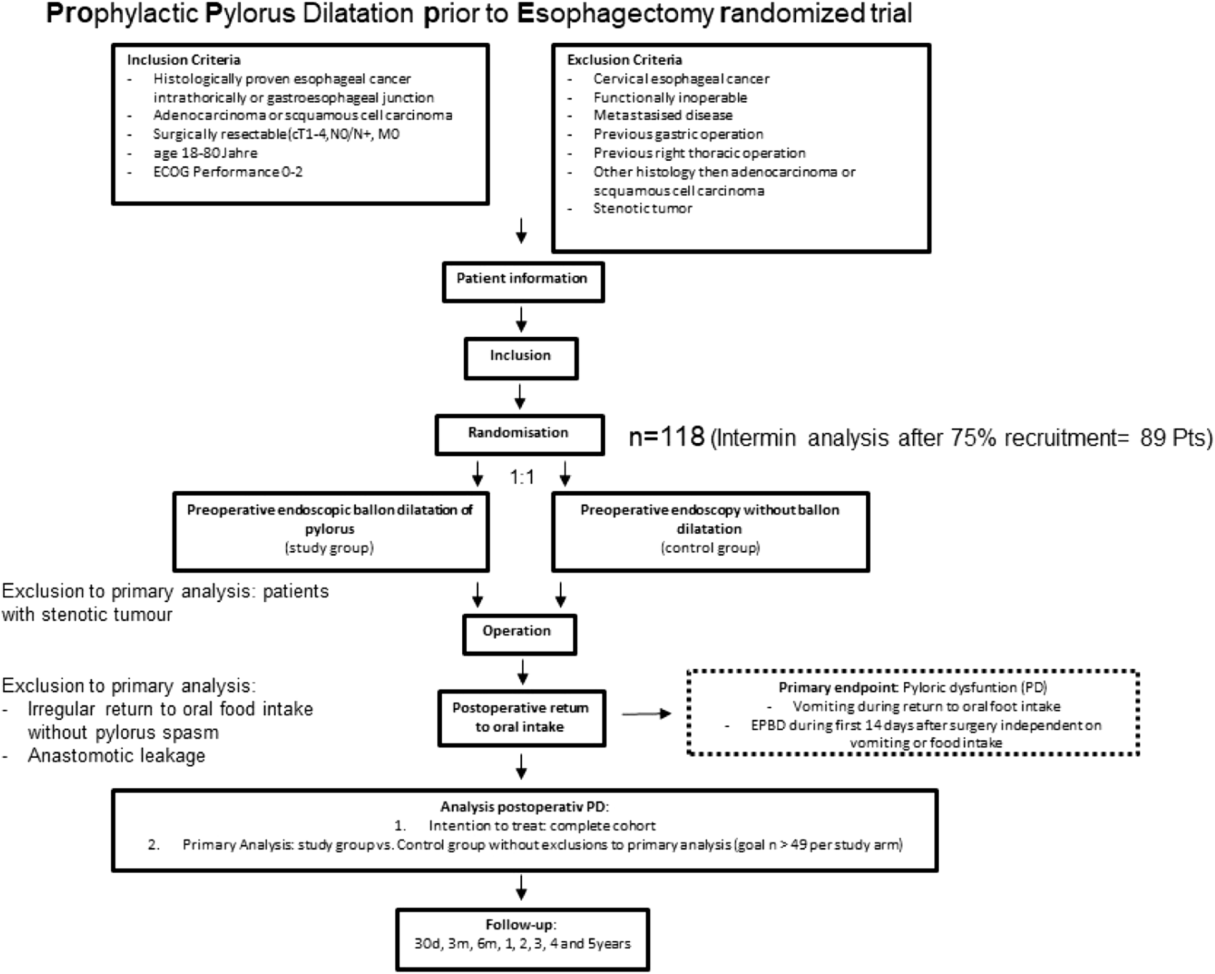
PROPPER trial flow chart

If any statistical method is needed to account for missing data, multiple imputations will be used. If necessary, professional help with the statistical evaluation is sought (Fig. 1).

### Follow-up and data management

Data collected during the hospital stay consists of the primary endpoint DGE rate as well as the secondary outcomes postoperative complication rates, in-hospital mortality, events during gastroscopy, length of ICU and hospital stay. All patients will be monitored for a follow-up period of 5 years. The follow-up intervals in the outpatient clinic will be 30 days, 3 months, 6 months, and 9 months in the first year, followed by biannual visits. Postoperative pain evaluation and quality of life scores will be collected preoperatively, 1 month, 6 months, and up to 5 years postoperatively. Postoperative pain will be assessed daily during hospitalization. All adverse events will be registered and collected in a patient file. All reports, collected data, processes, and administration forms will be encrypted by a coded identification number (ID) to maintain participant confidentiality. The coding list for potential decryption can exclusively be accessed by the study coordinator and the principal investigator. The local database will be secured by a password-protected access system. All files concerning participants (containing names or other personal identifiers) will be stored in a separate locked file in an area with limited access. All data between centers involved in the study will be pseudonymized. All data will be archived for 10 years after the end of the study. An internal audit of the study will be carried out annually in the study center. Table 2 shows the time schedule of enrollment, interventions, and assessments.

**Table 2:** Schedule of enrollment, interventions, and assessments

Timepoint	Study period						
	Screening/allocation	Post-allocation					End of FU
	D − 1: Outpatient clinic visit	D0: 1 day pre OP	D1: hospital discharge	Hospital discharge	D30: 30 day post OP	D90: 3-month post OP	5 years
**Enrollment:**							
Eligibility screen	X						
Informed consent	X						
Allocation	X						
**Interventions:**							
Gastroscopy without EPBD		X					
Gastroscopy with EPBD		X					
**Assessment:**							
Baseline characteristics	X						
DGE (primary outcome)			X	X	X	X	
Secondary outcomes^a^			X	X	X	X	
QoL Questionnaire	X		X		X	X	
Pain score	X		X		X	X	
Overall survival			X	X	X	X	X

*FU* Follow up, *DGE* delayed gastric emptying

^a^Secondary outcomes include major and minor postoperative complication rates, in-hospital mortality, adverse events during gastroscopy, length of ICU, and hospital stay

### Data analysis

The statistical analysis will be performed using the statistical program SPSS (IBM). Both an intention-to-treat analysis and per-protocol analysis are planned. Primary and secondary outcomes will be compared using the chi-squared test, Fisher exact test for categorical variables, and Mann–Whitney *U* test for non-parametric variables. Mortality will be compared using Kaplan–Meier curves and log-rank test. The pain scales will be analyzed using mixed linear models, quality of life by using an analysis of covariance.

There will be an interim analysis after 75% of the participants were monitored for the occurrence of the primary endpoint. If the hypothesis is confirmed or reaching statistical significance is impossible at that point, the study will be ended.

### Dissemination plans

The trial results will be presented at scientific conferences and published in appropriate medical journals. The present article is the trial protocol. Patients may request a summary of the trial results through the hospital website or directly from the study team.

## Discussion

Postoperative DGE after esophacetomy for cancer is a common postoperative complication that can lead to increased rates of pneumonia and cardiac complications [4].

Apart from pyloric dilatation, alternative intraoperative procedures have been suggested and investigated by different authors. Surgical pyloroplasty had potential effects on postoperative DGE rates in retrospective studies [10, 20]. However, a systematic review published in 2015, including 3172 patients, revealed only a nonsignificant effect of pyloric drainage on postoperative DGE rates [13]. Additionally, the effect seems rather small, considering the additional procedure steps and potential complications arising from this intervention. Some authors reported increased rates of biliary reflux and dumping syndromes, which can result in significant postoperative morbidity [21, 22]. The results from chemical pyloroplasty with injection of botulinum toxin have been disappointing; in the meta-analysis from Nienhüser et al., DGE was not affected by injection of botox (OR 0.87, 95% confidence interval (CI) 0.37–2.03, *P* = 0.75).

In contrast to the above-mentioned procedures, preoperative EPBD offers promising results while avoiding invasive procedures with possible side effects. So far, the effect of EPBD on postoperative rates of DGE has only been analyzed in retrospective studies and case series [14, 23], suffering from bias regarding patient selection as well as small sample sizes. Nienhüser et al. included four retrospective studies analyzing the effect of mechanical pyloric stretching on postoperative DGE. First, in 2010, Deng et al. presented 48 patients in which manual pylorus dilatation was performed during surgery and compared those to a placebo group. They found significantly higher rates of DGE (13.3% vs. 0%) in the non-dilatation group measured by gastric scintigraphy on postoperative day 14 [24]. In 2014, Antonoff et al. published their study including 8 patients in which digital dilatation was performed. None of those experienced DGE [22]. Boshier et al. used an intraoperative method for pylorus dilatation in their study in 2018. They analyzed 100 patients; half of them received a pylorus dilatation using a forceps after entering through a gastrostomy. Those patients had a significantly lower rate of DGE (22% vs. 48%, *p* = 0.006) [25]. These studies, as well as the presented study from Hadzijusufovic et al., suggest that preoperative or intraoperative mechanical pylorus stretching might be an effective prophylactic procedure to prevent postoperative DGE. However, the above-mentioned studies were conducted in the era of open esophagectomy. Manual pylorus stretching can not be carried out easily during minimally invasive procedures without adding additional steps. Therefore, preoperative or intraoperative endoscopic dilatation is the most feasible approach for pyloric stretching in minimally invasive procedures. However, as mentioned in the introduction, the retrospective study from Hadzijusufovic et al. has several limitations, especially regarding patient selection bias as well as lack of blinding. The presented PROPPER trials try to address this evidence gap and provide evidence-based insight on the efficacy of preoperative EPBD on postoperative DGE rates. If proven effective, the results of this trial could influence treatment guidelines for esophageal cancer surgery and postoperative complications such as pneumonia resulting from DGE could be avoided.

There are some limitations of this study. First, DGE is not defined clearly in the literature. Some studies use imaging methods such as upper GI water-soluble contrast radiogram for definition. However, this diagnostic modality has a high risk of aspiration and therefore was abolished in the international expert consensus as a recommended diagnostic criterion [16]. In this study, vomiting was used as a parameter of the primary outcome since the main focus was set on the patients’ experience and the patients’ symptoms mostly decide over the indication of postoperative interventions.

Additionally, the study was planned with a limited sample size. We used the retrospective study published by Hadzijusufovic et al. because in their study the same technique was used as it is in the PROPPER trial. DGE can be influenced by many different disturbance variables, such as anastomotic technique, nerve sparing techniques, postoperative return to oral intake, conduit size or reconstructive route. Therefore, the intention of the PROPPER trial was to keep the techniques and perioperative pathways as homogeneous as possible. In the three participating centers, the techniques of the study are carried out appropriately, as all surgeons took part in a rotation program and were trained by the same specialist. Furthermore, a higher sample size would increase the recruiting time, which would risk other technique changes over time that could bias the outcome (e.g., anastomotic technique changes, different robotic approaches). The sample size was calculated on the basis of the preexisting evidence.

## Conclusion

The presented PROPPER trial is a randomized controlled trial aiming to provide more evidence regarding the prevention of delayed gastric emptying by prophylactic endoscopic pyloric dilatation prior to esophagectomy for cancer.

## Supplementary Information

Supplementary Material 1.

## Data Availability

The study data will be available upon reasonable request to the corresponding author.
